# Illness and treatment beliefs in Kathmandu valley Nepalis under hypertensive care

**DOI:** 10.1371/journal.pgph.0004270

**Published:** 2025-06-12

**Authors:** Deepak S. Shrestha, Andrea M. Straus, Ram Kishor Sah, Hari Har Khanal, Roshani Gautam, Bishnu Dutta Paudel, Talea M. Cornelius, Richard R. Love

**Affiliations:** 1 Peoples Dental College and Hospital, Kathmandu, Nepal; 2 Amader Gram, Rampal, Bangladesh; 3 Civil Service Hospital, Kathmandu, Nepal; 4 Lalitpur Heart Clinic, Lalitpur, Nepal; 5 Nursing Campus Maharajgunj, Institute of Medicine, Kathmandu, Nepal; 6 KIST Medical College, Lalitpur, Nepal; 7 Columbia University Medical Center, New York City, New York, United States of America; Oxford University Clinical Research Unit Nepal, Patan Academy of Health Sciences, NEPAL

## Abstract

The prevalence of actionable (i.e., stage 2 = BP > 140/90) hypertension among Nepalis approaches one-third in the adult population. Long-term drug treatment adherence is low. Western-developed assessment tools present an incomplete picture of the regional Nepali beliefs that guide patient treatment adherence, and the present study seeks to substantiate qualitative findings that suggest a reorientation of patient education content for more effective patient-oriented, culturally consonant care. We conducted a cross-sectional structured interview of 500 men and women under treatment for hypertension in three urban Kathmandu hospital clinics. Interview items were developed from a qualitative survey and investigated patient beliefs about hypertension causes, symptoms, and consequences; treatment costs and sequelae; and general health maintenance. Patients reported experiencing multiple and wide-ranging symptoms that affected how they felt physically and emotionally as well as how they behaved; these were expected to respond to treatment. They were distressed by their condition and were concerned about short- and long-term treatment effects, specifically that long-term treatment may impair the body’s natural ability to maintain a healthy blood pressure. While hypertension was described as life-long, there was a range of beliefs on whether successful blood pressure control would, or should, allow a switch to natural remedies, lifestyle management, or no treatment at all. Kathmandu Valley hypertension beliefs suggest adaptation of Western-developed patient communications to address Nepali patient experience and concerns. While Western communications address the “silent” nature of the disease, our Kathmandu population experienced multiple and varied symptoms that guided their behavior. Patient also expressed concerns about medication habituation and dependence reflecting Nepali health models.

## Introduction

The prevalence of actionable, i.e., stage 2 = BP > 140/90, hypertension among Nepalis approaches one-third in the adult population and may be increasing [1–5]. Despite the availability of effective and inexpensive drugs, long-term treatment adherence is poor and population reductions in the morbidity and mortality associated with hypertension are not being observed [5,6]. Nonadherence to hypertensive drug treatment was found to be approximately 50% at the time of assessment in two Nepalese studies, consistent with what is believed to be a challenging problem worldwide [7–9]. A Cochrane Report on treatment adherence suggested that there have been limited insights in recent years into this major issue [10]. In particular, there are limited data about the role of cultural models and social determinants in treatment adherence despite their significance for behavioral medicine [11].

Previously, the investigators conducted an exploratory study of patients under treatment for hypertension in the outpatient clinic of a tertiary-care institution in Kathmandu [12]. Hypertension was identified as having multiple, widely varying symptoms by 42 of the 50 patients interviewed, including behavioral and affective elements. It was also identified as a serious disease like other noncommunicable conditions. There were frequent expressions of concern about long-term medication as well as barriers to treatment. Patients appeared to be influenced by the holistic Ayurvedic and Yoga frameworks that emphasize wellness maintenance rather than “…the suppression of sickness with life-long consumption of drugs” [13]. Yoga practices and theory are part of school curricula and Ayurvedic medicine is a licensed mainstream practice that identifies illness as an imbalance in the basic forces underlying body, mind, and behavior. It emphasizes as well that long-term use of medicine will impair the body’s ability to maintain, in this case, blood pressure. [13,14]. We conducted observational cross-sectional interviews in a larger population to confirm these qualitative themes that suggest how we might adapt Western-developed patient communications to address Nepali misconceptions in the context of Nepali experience.

## Methods

### Ethics statement

This study was approved by the Ethics Committee of the Nepal Health Research Council I.D. # 622/ 2020 P on November 10, 2020, and subsequently by the Institutional Review Board at Marquette University in the United States. Additional information regarding the ethical, cultural, and scientific considerations specific to inclusivity in global research is included in the supporting information S1 Checklist. Formal written consent was obtained from all respondents.

### Study protocol

Observational cross-sectional interviews were conducted with 500 clinic patients over 18 years old who self-identified as hypertensive. No other exclusion criteria were employed. Interview items were developed from the themes and language identified in the English translations of our earlier qualitative investigation of Nepali beliefs about hypertension and its treatment [12], consistent with the phenomenological approach of our investigations. The interview instrument was translated into Nepali by the Nepali researchers relying on patient phrasing from the qualitative study, then back translated to English. In the absence of any knowledge about how beliefs may cluster in subgroups of this culturally diverse city, we chose a large sample.

Male and female interviewers were trained in structured interview technique as well as Leventhal’s “Common-Sense Model of Self-Regulation” (CSM), a widely used theoretical framework describing the dynamic process by which patients become aware of an illness, formulate an image of the threat and potential treatment, adopt action plans for addressing the threat, and integrate feedback on treatment efficacy and illness progression [15]. The interviewers did not present themselves as clinicians to avoid the impression that they were evaluating patients on their recall of doctor communications rather than their own beliefs.

Interviews were conducted at three hospital outpatient clinics with services provided self-pay (Table 1) from 05-04-2021 till 27-04-2021. Two are government facilities drawing from a diverse national population, although the majority of patients are from the Kathmandu Valley: The referral center Shahid Gangalal National Heart Center, Bansbari, Kathmandu and the tertiary facility National Academy of Medical Sciences’ Bir Hospital, Kathmandu. The third site, the Lalitpur Heart Clinic, Lalitpur, Kathmandu Valley, is a private clinic with somewhat higher fees and a more local catchment area.

**Table 1:** Patient demographics.

N = 500	
Interview site	n(%)
Galangal National Heart Center	245(49.0)
NAMS Bir Hospital	165(33.0)
Lalitpur Heart Clinic	90(18.0)
Gender	
Male	268(53.6)
Female	232(46.4)
Age in years	Mean = 57.1 SD = 11.3
First language	
Nepali	351(70.2)
Nepali and other	29(5.8)
Newari	81(16.20
Maithili	13(2.6)
Other	26(5.2)
Comorbidities, solicited	
Heart disease	131(26.2)
Diabetes	103(20.6)
Hyperlipidemia	73(14.6)
Kidney disease	22(4.4)
Stroke or paralysis	5(1.0)
No reported comorbidities	189(37.8)
Comorbidities, volunteered	
Thyroid conditions, gastritis, hyperuricemia, arthritis, and allergies	187(37.4)

In the outpatient clinics of these institutions, individuals who self-identified as hypertensive patients were recruited with a financial incentive of 600 rupees NPR, a little under $5.00 USD. Recruitment included description of the study protocol and duration, as well as assurance that participation would not delay their visit and nonparticipation would have no effect on clinic services. Ten percent of eligible male and female individuals declined study participation. Written informed consent was obtained from 268 male and 232 female participants. Interviews were conducted in Nepali in quiet private rooms and lasted approximately 30 minutes. Care was taken to avoid interfering with the clinic visit and standardized assessment tools were not included to curtail interview time. Following a brief set of open questions, patients indicated agreement with questionnaire items using a graphic 5-point bimodal Likert scale presented with instructions appropriate for literate and illiterate respondents. The complete interview schedule is provided in the supporting S1 file Protocol. Subjects’ demographic information and response record were entered into a computer database in English with the file identified by number only.

Patient responses were summarized by measures of central tendency and dispersion appropriate to their distribution. Using SAS, an analysis of K-means clustering was employed to identify naturally occurring groupings of these beliefs, and Pearson product-moment correlations with pair-wise deletions were conducted between items indexing concerns with disease and its treatment.

## Results

The interviewed patient sample (N = 500) is described in Table 1. The majority of patients came from the public hospitals that served the general Kathmandu Valley population, while 18% came from the more expensive private clinic. The preponderance of male respondents and the mean age of 57.1% were not unexpected in a sample of hypertensive patients as was the language mix. While patients were asked about a subset of comorbidities, other conditions were also volunteered.

Patients reported that hypertension not only affected their physical well-being, but also their emotions and their behavior (Table 2). Less than 10% reported that they could not tell when their blood pressure was high and those who reported being symptomatic had an average of 5.3 symptoms.

**Table 2:** Signs of high blood pressure reported by patients (N = 500).

High blood pressure sign	n(%)
Can’t tell when blood pressure is high	46(9.2)
Headache	327(65.4)
Dizziness or fainting	292(58.4)
Aggressive behavior	209(41.8)
Palpitations	186(37.2)
Tingling sensations in arms and legs	171(34.2)
Blurred vision, flashes of light	165(33.0)
Fatigue, lethargy, heaviness	124(24.8)
Feel hot, fever	115(23.0)
Sweating	110(22.0)
Fearfulness, panic	100(20.0)
Restlessness	87(17.4)
Insomnia	87(17.4)
Shortness of breath	75(15.0)
Body pain or aching	68(13.6)
Gastritis, nausea	63(12.6)
Unhappiness, depression	48(9.6)
Shivering	38(7.6)
Slurred speech, numb tongue	37(7.4)
Tinnitus	33(6.6)
Disoriented	20(4.0)
Ear pain	18(3.6)
Paralysis	17(3.4)

Many symptoms are ones associated with stage 3 hypertension [16] including the two most commonly reported, headache and dizziness or fainting, but also heart palpitations, tingling sensations in arms or legs, blurred vision or flashes of light, fearfulness or panic, shortness of breath, gastritis or nausea, tinnitus, feeling confused or disoriented, and ear pain. Other symptoms appeared less associated with high blood pressure in the medical literature, most prominently aggressive behavior, the third most common symptom, but also fatigue or lethargy, sweating, insomnia, restlessness, body pain, unhappiness or depression, shivering, slurred speech or numb tongue and even paralysis.

Majorities of patients rated high agreement with statements that they were symptomatic at diagnosis, that their high blood pressure had symptoms, and that they could tell when their blood pressure was high. A majority also reported that treatment should affect their symptoms although they pursued treatment to prevent serious future consequences (Table 3).

**Table 3:** Likert scores of patient health beliefs.

*Likert Rating Scale* * 1—---------------------------------------------------2—------------------3—------------------4—------------------5* *Disagree completely Neither agree nor disagree Agree completely*		
	Median	IQR
I can tell when my blood pressure is high	5	1
When my blood pressure is high, it causes symptoms	5	1
Hypertension is a serious disease	5	1
My illness makes me upset, angry, scared, or depressed	4	4
I expect that hypertensive medication will change or stop my symptoms	5	1
I will have hypertension for my whole life	5	2
Visiting the clinic or doctor every 3–6 months is necessary to manage my hypertension	5	0
I think that following the recommendations of my western medical doctor is the most important thing I can do for my health	5	0
I will have to take medication for the rest of my life	5	1
I take medication to prevent future health problems like heart disease, stroke, or kidney disease which are caused by hypertension	5	1
I am concerned about the difficulty of making healthcare visits for my hypertension	4	3
I am healthy when the right medicine controls my disease	5	1
My symptoms of hypertension are the reason I went to the doctor and was diagnosed	5	1
Hypertension belief (continued)	Mean	SD
My hypertension comes and goes	3.22	1.43
My hypertension can be cured and then I can stop taking treatment	2.62	1.52
My hypertension is caused by a family history of hypertension or other disease	2.55	1.59
My hypertension was caused by an imbalance I was born with	1.86	1.20
My hypertension is due to money, work, or family stresses	3.20	1.58
My hypertension is due to my diet, lack of exercise, smoking, or drinking alcohol	3.47	1.43
I think that balancing the different forces (doshas or sheaths) in my body is the most important thing I can do for my health	3.81	1.11
I think that my health reflects the type of person I am and my type of body	3.93	0.90
Once the medication has worked, I can stop it and use natural remedies or diet and exercise to control my hypertension	3.40	1.45
I only take medication when I think my blood pressure is high	1.79	1.13
I am concerned that the medication will affect my body’s ability to maintain balance and/or control blood pressure on its own	2.94	1.40
I am concerned about the side effects of medication at the present time	2.50	1.47
I am concerned about the long-term side effects of medication	2.92	1.52
I am concerned about the safety of taking medicines long-term for hypertension	3.38	1.51
I am concerned about the cost of medication at the present time	2.77	1.57
I am concerned about the cost of taking medication for the rest of my life	3.27	1.66

Patient temporal expectations for hypertension and its treatment were also assessed. Patients endorsed the beliefs that both their hypertension and their treatment are life-long and disagreed with medicating only when symptomatic. There was not as much agreement on whether hypertension comes and goes and whether the need for medication could be superseded by natural treatments or life-style change, or even stopped altogether (Table 3).

**Fig 1:**
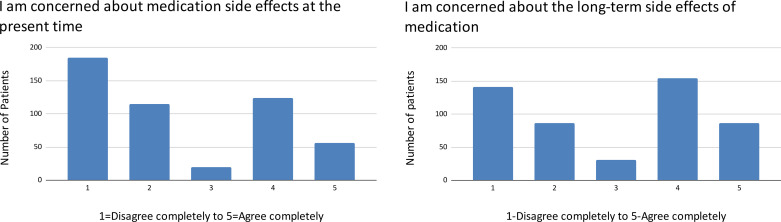
Concerns about medication side effects.

**Fig 2:**
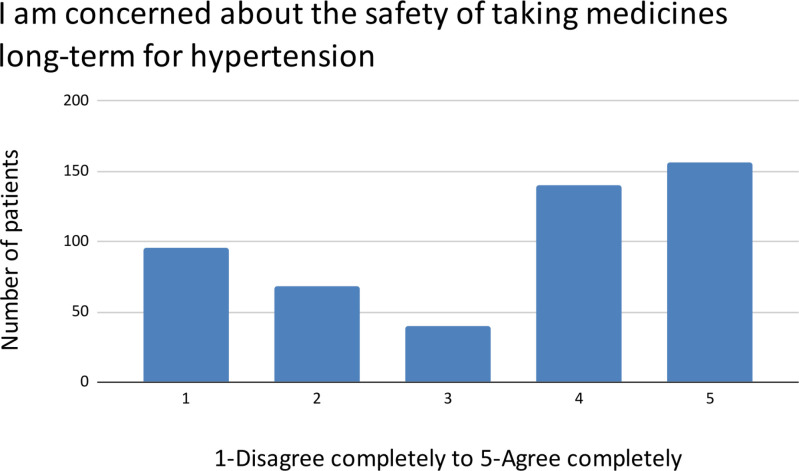
Concerns about long-term medication safety.

There is a bimodal distribution of patient concern about immediate and long-term side effects of medication (Fig 1) as well as the long-term safety of medication (Fig 2).

**Fig 3:**
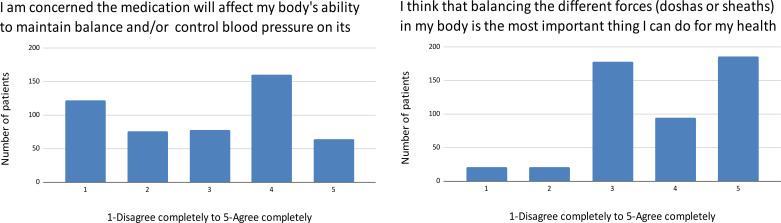
Ayurvedic belief and specific Ayurvedic terminology.

The Ayurvedic belief that long-term medication damages the body’s ability to control blood pressure received a spectrum of agreement, while the item employing Ayurvedic and Yoga terminology varied between “Neither Agree or Disagree” and “Agree completely” (Fig 3).

Pearson product-moment correlations were computed for the short- and long-term concerns about side effects and treatment safety as well as barriers to treatment (Table 4). Weak positive correlations are found among these concerns with the following exceptions:

**Table 4:** Correlations of medication concerns and barriers to treatment.

Concerns	Long-term side effect	Long-term safety	Damage to body’s balance	Current costs	Long-term costs	Clinic access
Current side effects	*r*(497)=.77 *p *< .001	*r(497)*=.43 *p *< .001	*r*(497)=.36 *p *< .001	*r*(497)=.25 *p *< .001	*r*(497)=.24 *p *< .001	*r(497)*=.27 *p *< .001
Long-term side effects		*r*(497)=.51 *p *< .001	*r*(497)=.40 *p *< .001	*r*(497)=.25 *p *< .001	*r*(497)=.26 *p *< .001	*r*(497)=.28 *p *< .001
Long-term safety			*r*(497)=.19 *p *< .001	*r*(497)=.12 *p *< .001	*r*(497)=.16 *p* < .001	*r*(497)=.19 p < .001
Damage to body’s balance				*r*(497)=.26 *p* < .001	*r*(497)=.23 *p* < .001	*r*(497) =.26 *p* < .001
Current costs					*r*(497)=.86 *p* < .001	*r*(497) =.54 *p* < .001
Long-term costs						*r*(497) =.52 *p* < .001

Short- and long-term concerns about side effects or costs of treatment have strong positive correlations. Both short- and long-term concerns with side effects have moderate positive correlations with the three measures of treatment barriers: short- and long-term costs and difficulties making healthcare visits. Between those three measures, both short- and long-term concerns with treatment costs have a moderate positive correlation with difficulty of healthcare visits.

**Fig 4:**
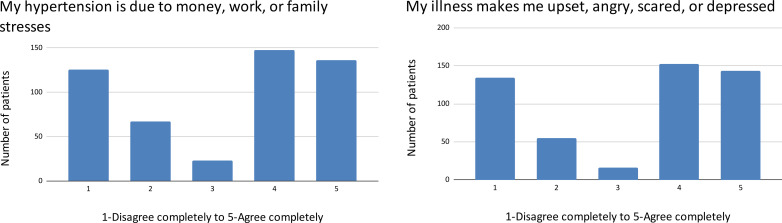
Emotional stress and hypertension.

Beliefs about the role of emotional distress as a cause and a consequence of hypertension produced a bimodal distribution (Fig 4).

**Fig 5:**
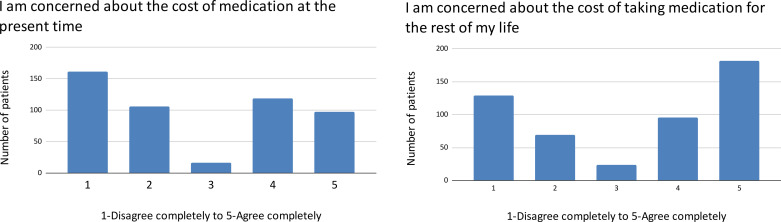
Financial barriers to care.

**Fig 6:**
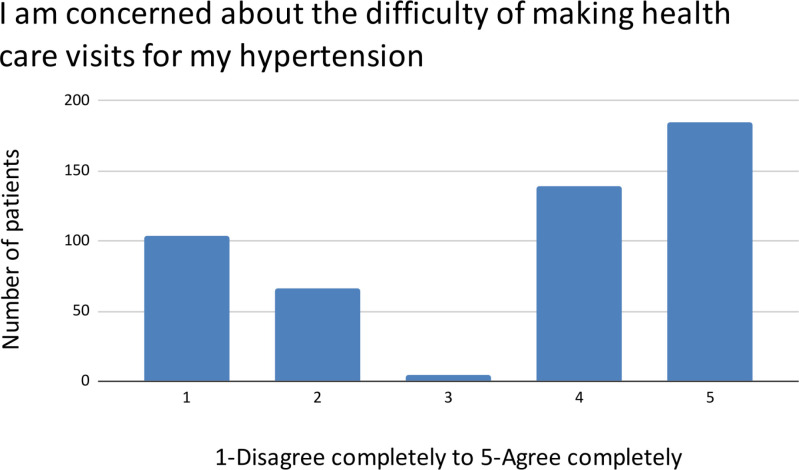
Treatment barriers.

A bimodal distribution is also found in concerns about the treatment barriers of current and future costs (Fig 5) as well as clinic accessibility (Fig 6).

The cluster analysis did not produce any interpretable patterns or groupings. The complete dataset is provided in S1 Dataset.

## Discussion

The wide range and large numbers of symptoms reported may be misattributed manifestations of other conditions, medication side effects, or even symptoms from high levels of hypertension. This divergence from the asymptomatic presentation in Western countries is also consistent with Nepali mainstream Ayurvedic and Yoga illness models, in which disease is a state of imbalance manifested in the whole person: body, mind, and behavior [12,13]. WHO and the American Heart Association emphasize that hypertension is a “silent disease” and promote detection and control in the *absence* of symptoms [16,17], but this characterization diverges so greatly from our population’s experience that greater consonance and therefore credibility may be achieved by focusing on the need to rely solely on blood pressure readings for assessing treatment effectiveness. This in turn requires access to blood pressure monitoring, if possible through the provision of blood pressure machines to patients themselves. Regular monitoring can demonstrate on the one hand that medication is effective even though “symptoms” are being experienced and on the other that blood pressure may be high in the absence of symptoms.

Patients were concerned that long-term medication would create a dependency or damage the natural ability to control blood pressure. While successful blood pressure control demonstrates the value of treatment to the clinician, it may signal the need to stop for the patient. Care should be taken to explain that treatment must be ongoing because hypertension is life-long and that medication supports the body’s ability to maintain wellness. Although these concerns with long-term treatment are consistent with Ayurvedic science, familiarity with the terms specific to that tradition were not as widespread and the belief should be addressed in colloquial language familiar to a wider population.

Patients also expressed concerns about treatment side effects, safety, and cost. The different treatment concerns have a highly significant *weak* correlation indicating that these concerns are not limited to a population segment generally anxious or fearful of medication. It is not enough to disseminate information on hypertension’s threats and the effectiveness of treatment - people also need to know that the treatment will be worth the costs, the barriers, and its potential damaging (side) effects. Crucial to this approach is encouraging clinicians to inform patients that side effects can be managed and to elicit and respond to reports of side effects and other treatment concerns.

Such clinical attention could also benefit the majority of patients who reported feeling upset, angry, scared or depressed by their hypertension. This might further be addressed in public health communications in which positive socially known figures who have (controlled) hypertension message that they have a serious disease, but they are calm and confident knowing they have their blood pressure under control with medication and healthy life habits. “I don’t need to worry because I take my medication and monitor my blood pressure.”

### Limitations

The data reported here are from Kathmandu Valley self-identified patients under clinical care. These individuals are likely to be better educated with greater financial resources and healthcare access than the majority of Nepalis, who live in ethnically distinct rural areas. Absent widespread screening, clinic consultation is typically scheduled in response to a symptom, therefore our population is more likely to consider themselves symptomatic. All patients were under treatment and thus the role of the observed beliefs in treatment abandonment could not be addressed, nor was patient blood pressure control, the ultimate goal, known.

## Conclusion

Kathmandu Valley hypertension beliefs suggest the need to adapt Western-developed patient educational materials to address Nepali patient experience and concerns. Clinical and public health communications that describe hypertension as a “silent” disease contradict the multiple wide-ranging symptoms experienced by our Kathmandu Valley population. Patient education might more profitably focus instead on the greater reliability of sphygmomanometer readings. The life-long need for medication should be clearly attributed to the disease’s nature and not medication habituation or dependence, with message reinforcement as blood pressure is brought under control. Individual clinical intervention should be supported by increasing access to medication and blood pressure monitoring.

The foregoing suggestions for patient-oriented and culturally appropriate public health and clinical communications should be rigorously tested in clinical trials. Similar investigations of patient representations of hypertension should be conducted in Nepal’s regional and rural populations with careful consideration of representative sampling to better guide communications about this condition.

## Supporting information

S1 Protocol Interview Instrument. (DOCX)

S1 Checklist Global ethical, cultural, and scientific inclusivity considerations. (DOCX)

S1 Dataset Complete data set. (XLSX)
