# Supplementary material for: Illness and treatment beliefs in Kathmandu valley Nepalis under hypertensive care
Source: PLOS Glob Public Health. 2025 Jun 12;5(6):e0004270. doi: 10.1371/journal.pgph.0004270 (PMC12161525; doi:10.1371/journal.pgph.0004270)
Supplement: S1 Protocol — (DOCX) [file pgph.0004270.s001.docx]

**Supporting information**

**Hypertension Beliefs Interview Instrument**  (translated from Nepali)

1. Site of interview

___NAMS, Bir Hospital, Kathmandu, Nepal

___Shahid Gangalal National Heart Center, Bansbari, Kathmandu, Nepal

___Lalitpur Heart Clinic, Lalitpur, Nepal

 2. Gender

___male

___female

3. DOB: ____________

4. First Language:

___Nepali

___English

___Maithili

___Other__________________

5. Do you have any of these other medical problems? (review list, check **all** that apply)

___Heart disease

___Diabetes

___Kidney disease

___Stroke or paralysis

6. Do you take doctor-prescribed medications every day other than your hypertensive medications?

___Yes     How many different medications do you take every day___________

___No

7. What specific activities are you doing now for your health other than medication? (check **all** that apply)

___Cleanliness

___Avoid salty foods

___Avoid oily foods

___ Avoid other specific foods which are ______________________________

___Include particular foods which are______________________________

___Timing my meals/eating

___Exercise other than Yoga

___Adequate rest and sleep

___Rest and sleep at the right times of day

___Avoiding stress/meditating/maintaining a calm mood

___Yoga

___ Control my weight

8. Where do you find information about how to keep healthy? (check **all** that apply)

___No source used

___TV

___Radio

___Newspaper

___Internet

___Facebook and other social media

___Family and friends

___Western medical doctor

___Ayurvedic doctor

___Other hospital or clinic staff (not a doctor)

___Medical post

___Medical shop

___Visiting volunteer or other home assistance persons

___Medical reports, literature

9. Where do you go to get your blood pressure measured? (check **all** that apply)

Name the specific place

___I can measure it at home

___I don’t have my blood pressure measured

10. What instructions have you been given by your doctor about taking your hypertensive medicines? (review list, check **all** that apply)

___Take the medicine every day

___Take the medicine when I have symptoms

___It would be harmful to my health to stop medication

___I was given no verbal instructions

___I was given no written instructions

___Once I start taking hypertension medicine, stopping it will cause problems

11. Which, if any, of the following instructions have you been given where you filled your prescription? (review list, check **all** that apply)

___Take the medicine every day

___Take the medicine when I have symptoms

___Once I start taking my medicine, stopping it will cause problems

___I was given no verbal instructions

___I was given no written instructions

12. What happens at a medical visit for your hypertension? (check **all** that apply)

____My blood pressure is taken

____I find out what my blood pressure measurement is

____I find out if my blood pressure is in the normal range or not

____My doctor asks me if I am taking my medication every day

____My doctor asks me if I believe there are side effects from my medication

____We talk about my concerns about my medication

13. People sometimes forget or don’t take their medication. Have you ever skipped taking hypertensive medication?

___Yes

___No

14.  What happened to cause you to skip your medicine (check **all** that apply)

___I never missed my medication

___I stopped 1-2 days because I forgot or ran out of meds by accident

 ___I stopped because I couldn’t afford it.

 ___I stopped because I could not get meds for reasons other than money.

 ___I stopped because my blood pressure wasn’t high and I didn’t need it

 ___I stopped because I was using another method to control my blood pressure

___ Other

15. How do you help yourself to remember when to take your medicine? (check **all** that apply)

___I just remember with no reminder

___With a meal

___Same time every day

___My family member reminds me

___When I wake up

___When I go to sleep

___When my blood pressure is high

___Other

16. Are there other specific things for your health that you do regularly **every day**?

___Yes

___No

17. What do you do? (check **all** that apply)

___I don’t have other regular daily health activities

___I eat particular foods

___Take medication for other conditions

___Exercise

___Meditate, do yoga

___Other

18. How can you tell when your blood pressure is high? (check **all** that apply)

___Headache ___ I can’t tell

___Dizziness or fainting

___Ear pain

___Tinnitus

___Blurred vision, flashes of light

___Slurred speech, numb tongue

___Tingling sensations in arms and legs

___Body pain or aching

___Gastritis, nausea

___Feel hot, fever

___Sweating

___Shivering

___Fatigue, lethargy, heaviness

___Paralysis

___Shortness of breath

___Restlessness

___Unhappiness, depression

___Insomnia

___Palpitations

___Disoriented

___Fearfulness, panic

___Aggressive behavior

(Instruct patient on use of 5-point scale)

19. I can tell when my blood pressure is high.

20. When my blood pressure is high, it causes symptoms.

21. Hypertension is a serious disease.

22. My illness makes me upset, angry, scared or depressed.

23. I expect that hypertensive medication will change or stop my symptoms.

24. I will have hypertension for my whole life.

25. My hypertension comes and goes.

26. My hypertension can be cured and then I can stop taking treatment.

27. My hypertension is caused by a family history of hypertension or other disease.

28. My hypertension is caused by an imbalance I was born with.

29. My hypertension is due to money, work, or family stresses.

30. My hypertension is due to my diet, lack of exercise, smoking, or drinking alcohol.

31. Visiting the clinic or doctor every 3-6 months is necessary to manage my hypertension.

32**.** I think that balancing the different forces (doshas or sheaths) in my body is the most important thing I can do for my health.

33. I think that my health reflects the type of person I am and my type of body.

34. I think that following the recommendations of my western medical doctor is the most important support for my health

35. Once the medication has worked, I can stop it and use natural remedies or diet and exercise to control my hypertension.

36. I will have to take medication for the rest of my life.

37. I only take medication when I think my blood pressure is high.

38. I am concerned that the medication will affect my body’s ability to maintain balance and/or control blood pressure on its own.

39. I take medication to prevent future health problems like heart disease, stroke or kidney disease which are caused by hypertension.

40. I am concerned about the side-effects of medication at the present time.

41. I am concerned about the long-term side effects of medication.

42. I am concerned about the safety of taking medicines long-term for hypertension.

43. I am concerned about the cost of medication at the present time.

44. I am concerned about the cost of taking medication for the rest of my life.

45. I am concerned about the difficulty of making health care visits for my hypertension.

46**.** I am healthy when the right medicine controls my disease

47. My symptoms of hypertension were the reason I went to the doctor and was diagnosed
