# Supplementary material for: Illness and treatment beliefs in Kathmandu valley Nepalis under hypertensive care
Source: PLOS Glob Public Health. 2025 Jun 12;5(6):e0004270. doi: 10.1371/journal.pgph.0004270 (PMC12161525; doi:10.1371/journal.pgph.0004270)
Supplement: S1 Checklist — (DOCX) [file pgph.0004270.s002.docx]

Inclusivity in global research

**Ethical considerations, permits and authorship**

*This section is applicable to all research types.*

Provide details as to who granted permissions and/or consent for the study to take place in the Methods section of your manuscript. This should include the names of **all** ethics boards, governmental organizations, community leaders or other bodies that provided approval for the study. If individuals provided approval refer to these people by their role or title but do not list their name(s).

This study was approved by the Ethics Committee of the Nepal Health Research Council I.D. # 622/ 2020 P on November 10, 2020, and subsequently by the Institutional Review Board at Marquette University in the United States.

Reported on page number: 8

If there were any deviations from the study protocol after approval was obtained please provide details of these changes in the Methods section of your manuscript.
Did this study involve local collaborators that are residents of the country where the research was conducted or members of the community studied? If you do not have any authors from said communities, please provide an explanation for this below.

Yes,

the following authors are Nepali residents providing medical services in the Kathmandu Valley: Deepak S. Shrestha at the Peoples Dental College and Hospital, Ram Kishor Sah at the Civil Service Hospital, Hari Har Khanalat the Lalitpur Heart Clinic, Roshani Gautam at the Nursing Campus Maharajgunj, Institute of Medicine, and Bishnu Dutta Paudel at the KIST Medical College. Reported on title page.

There were no deviations from study protocol after approval.

Everyone listed as an author should meet PLOS’ criteria for authorship and all individuals who meet these criteria should be included in the author byline, rather than the acknowledgements. For further information please see the journal’s Authorship Policy.

**Human subjects research (e.g. health research, medical research, cross-cultural psychology)**

Did you obtain written informed consent from a representative of the local community or region before the research took place? How did you establish who speaks for the community? Details of written informed consent obtained from study participants should be reported separately in the Methods section of your manuscript.

Approval by the Ethics Committee of the Nepal Health Research Council was conditional on consent by the clinical sites at which patients were recruited. Patients were fully informed verbally and in written form before consent. including the absence of any consequences for declining to participate. The specifics of the consent protocol are on page.

How did members of the local community provide input on the aims of the research investigation, its methodology, and its anticipated outcome(s)?

Both the language employed and the topics covered in the interview instrument were observed in a qualitative investigation in the same population. Patients expressed concerns in that earlier study about medication and about barriers to care, and shared their beliefs about hypertension. Their observations as well as the language used to convey them are employed in the current investigation.

When engaging with the local community, how did you ensure that the informed consent documents and other materials could be understood by local stakeholders?

Nepali is the official language of Nepal and the language in which medical care in the study’s clinical settings is provided. Interviewers attended to patient comprehension when approaching potential subjects in the clinics and reported no communication difficulties.

Will the findings of the research be made available in an understandable format to stakeholders in the community where the study was conducted (e.g. via a presentation, summary report, copies of publications, etc.)? Please provide details of how this will be achieved.

Recommendations based on our descriptive findings are integrated into the authors’ facilities by presentations to the clinical staff. Further dissemination in medical educational facilities awaits publication in your peer-reviewed journal. While patient populations will not receive a formal report they will benefit from clinical communication that addresses their concerns and that is communicated in terms more consonant with their experience and beliefs, and therefore more easily understood and integrated into their beliefs and behavior.

**Non-human subjects research using specimens/ animals collected as part of the study, or those housed in archival collections. Examples include archaeology, paleontology, botany and zoology.**

Not applicable
